# Replication and Expression of the Consensus Genome of Hepatitis B Virus Genotype C from the Chinese Population

**DOI:** 10.3390/v15122302

**Published:** 2023-11-23

**Authors:** Fenfang Liao, Junmou Xie, Rongsong Du, Wenbo Gao, Lanyin Lan, Min Wang, Xia Rong, Yongshui Fu, Hao Wang

**Affiliations:** 1 Guangzhou Blood Center, Guangzhou 510091, China; fenfangweiyi@163.com (F.L.);; 2 The Key Medical Laboratory of Guangzhou, Guangzhou 510091, China

**Keywords:** Hepatitis B virus genotype C, HBV genome, consensus sequence, HepG2 cells, Huh7 cells, BALB/c, CD8, CD4, F4/80, Kupffer cell

## Abstract

Hepatitis B virus (HBV) genotype C is a prevalent HBV genotype in the Chinese population. Although genotype C shows higher sequence heterogeneity and more severe liver disease than other genotypes, its pathogenesis and immunological traits are not yet fully elucidated. In this study, we first established and chemically synthesized the consensus sequence based on representative 138 full-length HBV genotype C genomes from the Chinese population. The pHBV1.3C plasmid system, containing a 1.3-fold full-length HBV genotype C consensus sequence, was constructed for subsequent validation. Next, we performed functional assays to investigate the replicative competence of pHBV1.3C in vitro through the transient transfection of HepG2 and Huh7 cells and validated the in vivo function via a hydrodynamic injection to BALB/c recipient mice. The in vitro investigation revealed that the extracellular HBV DNA and intracellular replicative intermediate (i.e., pregenomic RNA, pgRNA) were apparently measurable at 48 h, and the HBsAg and HBcAg were still positive in hepatoma cells at 96 h. We also found that HBsAg and HBeAg accumulated at the extracellular and intracellular levels in a time-dependent manner. The in vivo validation demonstrated that pHBV1.3C plasmids induced HBV viremia, triggered morphological changes and HBsAg- or HBcAg- positivity of hepatocytes, and ultimately caused inflammatory infiltration and focal or piecemeal necrosis in the livers of the murine recipients. HBV protein (HBsAg) colocalized with CD8^+^ T cells or CD4^+^ T cells in the liver. F4/80^+^ Kupffer cells were abundantly recruited around the altered murine hepatocytes. Taken together, our results indicate that the synthetic consensus sequence of HBV genotype C is replication-competent in vitro and in vivo. This genotype C consensus genome supports the full HBV life cycle, which is conducive to studying its pathogenesis and immune response, screening novel antiviral agents, and further optimizing testing and therapeutics.

## 1. Introduction

Hepatitis B virus (HBV) infection is a global public health problem [1,2]. It causes inflammation of the liver tissue, which may result in liver cirrhosis and hepatocellular carcinoma (HCC) [3]. It is estimated that approximately 800,000 deaths per year could be attributed to the consequences of acute or chronic HBV infection [4]. In consideration of the high burden of HBV infection, the Global Health Sector Strategy to eliminate viral hepatitis by 2030 was approved at the 69th World Health Assembly [5]. The prevalence of hepatitis B surface antigen (HBsAg) in China declined to 5–6% in 2016 [6], which could be attributed to the policy of prophylactic vaccine. Nevertheless, China still faces challenges in achieving its goal of a 65% reduction in mortality from HBV by 2030 [7].

HBV—a member of the *Hepadnaviridae* family with a narrow host range and hepatotropic feature—is a small DNA virus with a 3.2 kb partially double-stranded circular DNA genome. The genome is composed of four overlapping open reading frames (ORFs: P, preC/C, preS/S, and X), four promoters (core promoter, CP; PreS1 promoter, SP I; PreS2 promoter, SP Ⅱ; and X promoter, XP), and two enhancers (Enhancer I, EN I; and Enhancer Ⅱ, EN Ⅱ), as well as a polyadenylation [poly(A)] signal. The ORFs are responsible for the encoding of seven distinct proteins (polymerase, HBeAg, HBcAg, L/M/S-HBsAg, and HBx) with the regulation of promoters and enhancers [8]. Biomarkers provide information on the replication of HBV. Specifically, HBV pregenomic RNA (pgRNA), which is packaged inside of the core particle for conversion to progeny DNA, is the most important HBV transcript required for viral reverse transcription and replication; HBV DNA, the only marker during the window phase of HBV infection, can be used to confirm viremia with HBsAg mutations; HBsAg, which makes the envelope of HBV, indicates the phase of infection; HBeAg, secreted from infected hepatocytes, is the marker of active virus replication; and HBcAg plays multiple roles in viral replication [4,9,10,11].

Based on an 8% or more divergence in HBV genome sequences, HBV has been classified into nine genotypes (A–I), with a 10th putative genotype (J) isolated from an individual in Japan [10,12]. These HBV genotypes can be further classified into at least 35 subgenotypes, based on a 4–8% intergroup nucleotide divergence across the complete genome. The number of subgenotypes widely varies among the genotypes, and the genotypes also greatly differ in disease progression, response to therapy, and clinical outcomes [13,14,15]. The distribution of HBV genotypes considerably varies across different areas of China. Specifically, HBV genotype B is predominant in central southern areas; genotype C is predominant in eastern, northern, and northeastern areas; genotypes B and C are both dominant in southwestern areas; and the recombinant genotype C/D is predominant in northwestern areas [16,17]. Genotype C is one of the most prevalent HBV genotypes in the Chinese population [16,17,18]. According to Li et al. [16], the proportion of genotype C is 75.3% in northern areas and 57% in southern areas. Genotype C is divided into 17 subgenotypes [19,20]. All of the identified subgenotypes of genotype C, except for C16, have been reported in Chinese areas [16,20], and C2 is the primary subgenotype of genotype C identified in China [16,18]. The *adr* serological subtype is dominant in genotype C among the Chinese population [21]. Other studies have indicated that genotype C is a significant cause of chronic HBV infection [22], and it is associated with a higher risk of liver cirrhosis and HCC [23,24]. Patients infected with genotype C are less sensitive to interferon (IFN) therapy than patients with genotype B [15]. Previous studies have mainly focused on the wild genome from infected patients with HBV genotype C [25,26,27]. However, potential mutations in the wild HBV genome alter the expression of HBsAg [28,29]. Therefore, a consensus sequence of HBV genotype C would be a good choice for studying its pathogenesis and immunological traits. However, the consensus sequence of HBV genotype C has not yet been functionally validated.

Here, we first established the consensus sequence of genotype C based on 138 full-length HBV genotype C genomes randomly obtained from the Chinese population. The consensus genome was chemically synthesized and inserted into a eukaryotic expression vector, namely pHBV1.3C. Next, we validated the feasible replication and expression of pHBV1.3C both in vitro and in vivo. The replication-competent consensus sequence provides a convenient tool for understanding the pathogenesis and virus-related traits of HBV genotype C.

## 2. Materials and Methods

### 2.1. Consensus Sequence

A total of 2038 full-length HBV genomes of genotype C submitted between 2009 and 2019 from different areas of China were downloaded from the NCBI database. Among these genomes of HBV genotype C, we retrieved 128 genomes using a random sampling method. Another 10 genotype C genomes were screened from blood donors at the Guangzhou Blood Center. A maximum of 10 genomes were obtained from a single author to ensure the representativeness of the consensus sequence. All 138 genomes were analyzed using BioEdit 7.0 software for entropy data and MegAlign 7.1 software for the consensus sequence. A single nucleotide of the consensus sequence was selected from the most frequent nucleotide at the same position (Supplementary Materials Table S2). Subsequently, phylogenetic analysis of the 138 full-length genomes with HBV genotype references or subgenotype C references was performed using MEGA 7.0 software, as described previously [30].

### 2.2. Plasmid Construction

The 1.3-fold consensus sequence of HBV genotype C (nucleotide 1038-3215/1-1984) was chemically synthesized (Sangon Biotech, Shanghai, China) and double-digested with *Hin*d Ⅲ and *Eco*R Ⅰ (TAKARA, Beijing, China). Next, it was cloned into an identical digested pcDNA3.1(+) (Sangon Biotech, Shanghai, China) vector, as previously described [28]. The new infectious plasmid, namely, pHBV1.3C, which contained a 1.3-fold consensus sequence of HBV genotype C, was subsequently constructed. The research was performed in accordance with the health guidelines related to recombinant DNA.

### 2.3. Cell Culture and Transfection

The HepG2 cells and Huh7 cells (Biospecies, Guangzhou, China) were cultured in Dulbecco’s Modified Eagle Medium (DMEM, Gibco Life Technologies, Grand Island, NY, USA), supplemented with 10% fetal bovine serum (FBS, Gibco Life Technologies, Grand Island, NY, USA) containing 100 units/mL of penicillin and 100 μg/mL of streptomycin (Gibco Life Technologies, Grand Island, NY, USA). The cells were kept at 37 °C in a humidified incubator with 5% CO_2_. For the in vitro studies, HepG2 cells and Huh7 cells were seeded in 12- or 24-well plates at an appropriate density to adhere overnight. The transient plasmid transfection of pHBV1.3C was performed, as per the manufacturer’s instruction, using Lipofectamine 3000 reagent (Invitrogen, Carlsbad, CA, USA). In this research, pHBV1.3B (constructed as previously described) [28] and pcDNA3.1(+) were used as the positive treatment and the mock treatment, respectively. At the indicated time, the cells and cell culture supernatants were harvested for different experiments.

### 2.4. Quantitative Analysis of Extracellular HBV DNA and Intracellular HBV pgRNA

For the analysis of extracellular HBV DNA and intracellular HBV pgRNA, HepG2 cells or Huh7 cells were seeded in 12-well plates in advance. At 5 h post-transfection, the cells were gently washed five times with 1× phosphate-buffered saline (PBS) to eliminate the influence of the transfected plasmids. At 48 h post-transfection, the cell supernatants were collected for HBV DNA analysis by quantitative real-time polymerase chain reaction (qPCR, fluorescence probe method) using an HBV Nucleic Acid Detection Kit (Daan, Guangzhou, China). The qPCR assays were performed in accordance with the manufacturer’s instructions. Total RNA was extracted from the 48 h-transfected cells using a Quick-RNA Purification Kit (ES science, Shanghai, China) for the analysis of intracellular HBV pgRNA. HBV genotype C pgRNA and β-actin in the HepG2 cells or Huh7 cells were quantified with the corresponding primers (HBV RNA: 5′-CTGGGTGGGAAGTAATTTGG-3′ and 5′-TAAGATAGGGGCATTTGGTG-3′; β-actin: 5′-TCCCTGGAGAAGAGCTACGA-3′ and 5′-AGCACTGTGTTGGCGTACAG-3′). The reverse transcription PCR and qPCR reactions were performed with a Fast All-in-One RT Kit (with gDNA Remover) (ES science, Shanghai, China) and a 2×Super SYBR Green qPCR Master Mix (ES science, Shanghai, China) as per the manufacturer’s instructions. The relative transcriptional folds were calculated as 2^−∆∆Ct^. The human housekeeper gene *β*-actin was used for expression normalization. These experiments were performed at least in triplicate. The qPCR was conducted using the applied biosystems of ABI 7500.

### 2.5. Immunofluorescence Staining

HepG2 cells and Huh7 cells were cultured for 96 h post-transfection with the plasmids in 24-well plates. Then, the cells were washed three times with 1×PBS. Immunofluorescence (IF) staining was carried out as described previously [28]. Briefly, HepG2 cells and Huh7 cells were fixed with 4% paraformaldehyde diluted using 1×PBS and permeabilized using 0.25% Triton X-100. After blocking with 1% bovine serum albumin (BSA), the cells were incubated with anti-HBsAg (ab68520, Abcam, Cambridge, UK) (1:500) and anti-HBcAg (ab8638, Abcam, Cambridge, UK) (1:500) antibodies containing 0.1% BSA at 4 °C overnight. The goat anti-rabbit IgG (H + L) CY3-conjugated (Affinity Biosciences, Changzhou, China) and goat anti-mouse IgG (H + L) Fluor488-conjugated (Affinity Biosciences, Changzhou, China) antibodies in a 1:200 dilution were used as the secondary antibodies. 4′,6-diamidino-2-phenylindole (DAPI) (Beyotime, Shanghai, China) was used to stain the nuclei. The cells were captured by fluorescent microscopy (Leica, Wetzlar, Germany) at a magnification of ×400.

### 2.6. Quantification Assays for HBsAg and HBeAg

The culture supernatants and cell lysates of the pHBV1.3C-transfected cells were separately obtained from 12-well plates at the indicated time points. Subsequently, the extracellular and intracellular levels of HBsAg and HBeAg were assessed using electrochemiluminescence (ECL) assays with Elecsys HBsAg II quant II (the lower limit of detection is 0.05 IU/mL) (Roche, Mannheim, Germany) and Elecsys HBeAg (the cutoff ≥1.0 is positive) (Roche, Mannheim, Germany) in accordance with the manufacturer’s directions. These experiments were performed at least in triplicate.

### 2.7. Hydrodynamic Injection and In Vivo Assays

Mice care and experimental procedures were carried out in accordance with national and institutional policies for animal health and well-being. The use of animals (six mice per group) for this study was approved by the Animal Management Committee of Guangzhou Blood Center. Male BALB/c mice [31] (6 to 8 weeks old, 16–22g) were purchased from Guangdong Medical Laboratory Animal Center. The plasmids of pHBV1.3C (30 μg, diluted with 1×PBS) were delivered into the mouse liver via a hydrodynamic injection (HDI) through the tail vein, and a pcDNA3.1(+) (30 μg, diluted with 1×PBS) injection was used as the negative control. The total volume, at 10% percent of the mouse weight, was delivered within 5–8 s. At the indicated time, blood samples were collected through the orbital cavity. The mice were euthanized to collect hepatic tissue samples.

For viremia analysis, the mice serum samples were separated and diluted at a ratio of 1:20 to assess the expression of HBsAg (Elecsys HBsAg II quant II) and HBeAg (Elecsys HBeAg) as mentioned above. Then, qPCR was performed for serum HBV DNA analysis as described above. The activity of alanine aminotransaminase (ALT, the value >50 IU/mL is positive) was measured with an ALT reagent (Kehua, Shanghai, China) as per the manufacturer’s instruction.

Hematoxylin–eosin (HE) staining and IF staining were performed for the histopathological and immunological analyses. Briefly, the hepatic tissues were fixed with 10% formalin and embedded in paraffin. The HE staining was conducted in accordance with the standard methods. For the IF staining, 5-μm-thick hepatic tissue sections were rehydrated and boiled for antigen retrieval. Then, the sections were blocked and incubated with the primary antibodies HBsAg (1:1000, Abcam, Cambridge, UK), HBcAg (1:500, Abcam, England), CD8 (1:500, Servicebio, Wuhan, China), CD4 (1:2500, Servicebio, Wuhan, China), and F4/80 (1:2500, Servicebio, Wuhan, China) at 4 °C overnight, followed by incubation with the corresponding secondary antibody and DAPI staining. The images were observed using Pannoramic DESK (3DHISTECH, Budapest, Hungary).

### 2.8. Statistical Analysis

The data, derived from three independent experiments, were exported by GraphPad Prism v7.0 (GraphPad Software, San Diego, CA, USA). The *p* values were obtained using the Mann–Whitney *U* test and a two-tailed unpaired Student’s *t*-test. Differences were considered significant at *p* < 0.05.

## 3. Results

### 3.1. Consensus Sequence of HBV Genotype C from the Chinese Population

**Figure 1:**
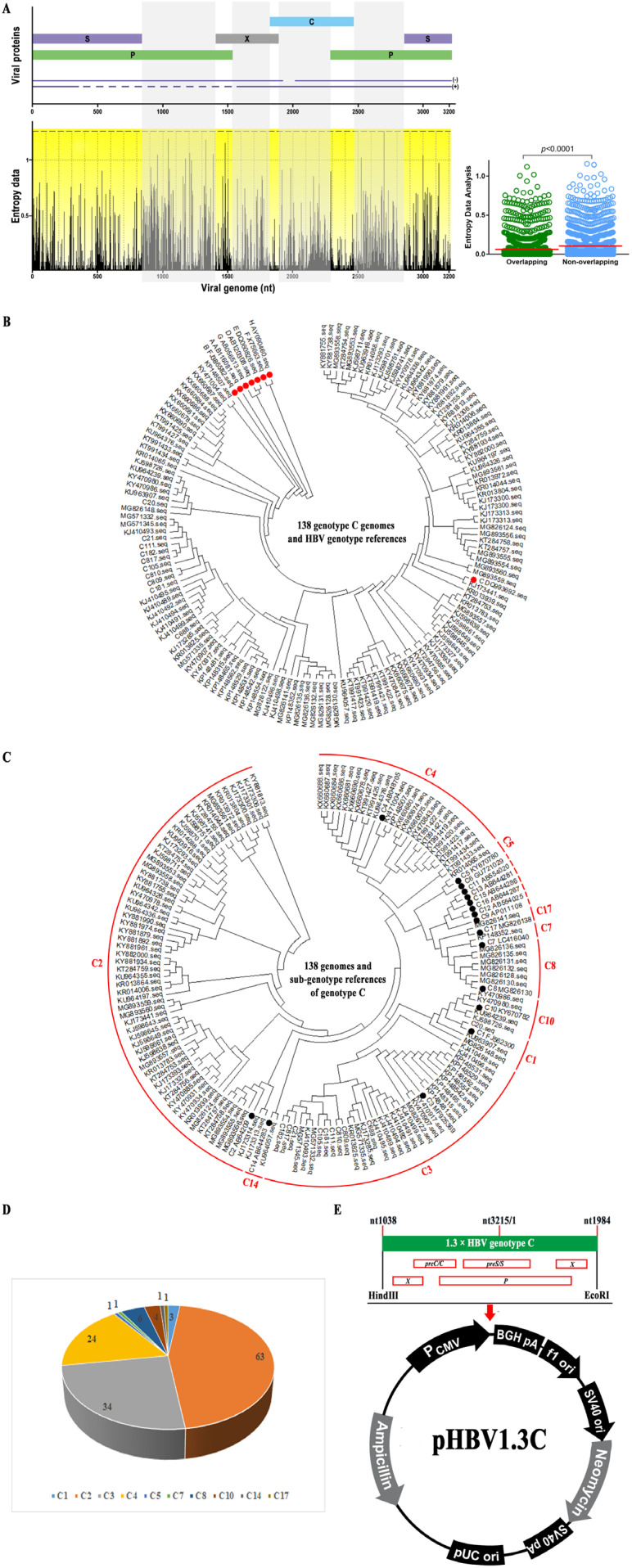
The annotation of the consensus sequence of HBV genotype C. (**A**) Variability distribution of the representative 138 HBV genotype C genomes from the Chinese population. Entropy analysis at each nucleotide within the dataset was performed using BioEdit 7.0 software. Mean entropy in the nonoverlapping regions was significantly higher at 0.105 (95% confidence interval, 0.097–0.114) than that in the overlapping regions at 0.062 (95% confidence interval, 0.055–0.068) (Mann–Whitney *U* test, *p* < 0.0001). (**B**) Phylogenetic tree of the relationship between 138 full-length genomes and HBV genotypes. The analysis was conducted based on the 138 full-length genomes of HBV genotype C. The phylogenetic tree was mapped with the neighbor-joining method. Red circle, the references of HBV genotypes. (**C**) Phylogenetic analysis of the subgenotypes among the 138 genotype C genomes. Black circle, the subgenotype references of genotype C. (**D**) The proportion of the subgenotypes among the 138 genomes of genotype C. (**E**) The infectious clone. The 1.3-fold full-length consensus sequence (nucleotide 1038-3215/1-1984) of HBV genotype C was synthesized and cloned into the pcDNA3.1(+) vector, namely pHBV1.3C, for functional validation.

Initially, 138 full-length genomes of HBV genotype C submitted between 2009 and 2019 in China were randomly obtained from the NCBI database. These genomes were aligned using MEGA 7.0. The whole-genome analysis of the 138 genomes indicated that the sequence variation was significantly higher in the nonoverlapping regions than in the overlapping regions by entropy analysis at each nucleotide (Figure 1A). Next, a comparison of all 138 full-length HBV genotype C genomes was performed using MegAlign 7.1 software to establish the consensus sequence (Supplementary Materials Figure S1), of which amino acid sequences from the four open reading frames were showed in Supplementary Materials Table S1. Genotype of the 138 genomes was verified through phylogenetic analysis, as described in a phylogenetic tree (Figure 1B). The subgenotypes of genotype C among the 138 genomes were further investigated by phylogenetic analysis (Figure 1C). We found that C2 was the major subgenotype (Figure 1D). Then, the 1.3-fold consensus sequence of HBV genotype C was synthesized and inserted into the *Hin*d Ⅲ and *Eco*R Ⅰ sites of pcDNA3.1(+) (Figure 1E). Finally, the infectious plasmid was named pHBV1.3C and used for the subsequent validation.

### 3.2. Replication of pHBV1.3C in HepG2 Cells and Huh7 Cells

**Figure 2:**
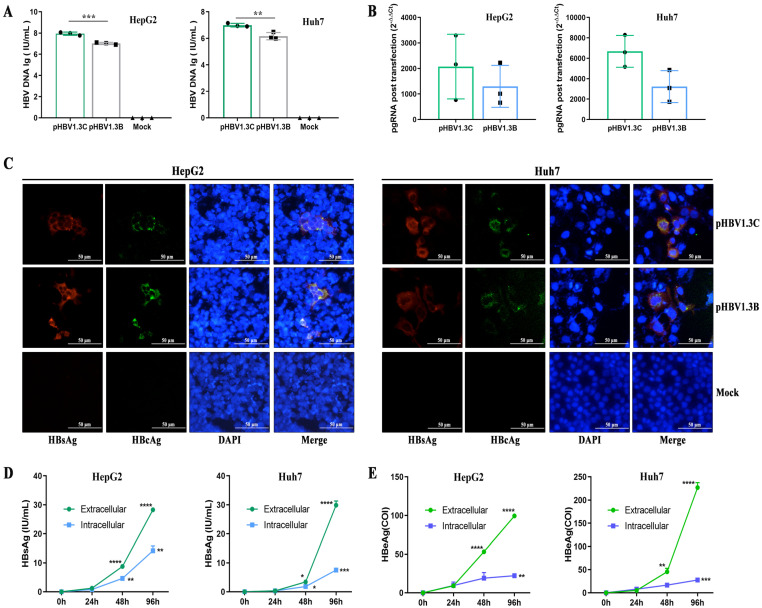
The replicative capacity of pHBV1.3C in HepG2 cells and Huh7 cells. (**A**) The extracellular HBV DNA level attributed to pHBV1.3C was analyzed by qPCR at 48 h. (**B**) The intracellular HBV pgRNA was examined by qPCR among the total RNA of the transfected cells at 48 h. Relative transcriptional folds were calculated as 2^−∆∆Ct^. The *β*-actin was used to normalize changes in pgRNA expression. (**C**) Visual detection of HBsAg and HBcAg in the transfected cells was performed using IF staining at 96 h (magnification ×400, scale bar 50 μm). Intracellular or extracellular HBsAg (**D**) and HBeAg (**E**) were estimated by ECL analysis of the transfected cell lysates and supernatants, respectively, at the indicated time points. Data are shown as mean values ± standard deviation, n = 3. A two-tailed, unpaired Student’s *t*-test was performed for *p* values vs. 24 h. * *p* < 0.05, ** *p* < 0.01, *** *p* < 0.001, **** *p* < 0.0001. pHBV1.3B, positive control; Mock, pcDNA3.1(+), negative control. The HBV biomarkers were undetectable in the mock treatment. lg: log10; pgRNA, pregenomic RNA; HBsAg, hepatitis B surface antigen; HBeAg, hepatitis B e antigen; HBcAg, hepatitis B core antigen; h, hours.

To examine the replication ability of pHBV1.3C in vitro, we analyzed the expression of HBV biomarkers after transiently transfecting pHBV1.3C into HepG2 cells and Huh7 cells. At 48 h post-transfection, the qPCR analysis indicated that the secreted HBV DNA was significantly raised in the pHBV1.3C cell supernatants relative to pHBV1.3B (the positive control), but undetectable in the mock treatment (Figure 2A); the pgRNA level of HBV was also increased in the pHBV1.3C-transfected cells (Figure 2B). Further, the IF staining revealed that HBsAg and HBcAg were still positive in the transfected cells at 96 h (Figure 2C). Moreover, the ECL analysis of the pHBV1.3C-transfected cells revealed that HBsAg (Figure 2D) and HBeAg (Figure 2E) accumulated in a time-dependent manner at both the extracellular and intracellular levels. These data suggested that pHBV1.3C was effectively replicated in the host hepatoma cells.

### 3.3. Kinetic Viremia of the BALB/c Recipients Induced by pHBV1.3C

**Figure 3:**
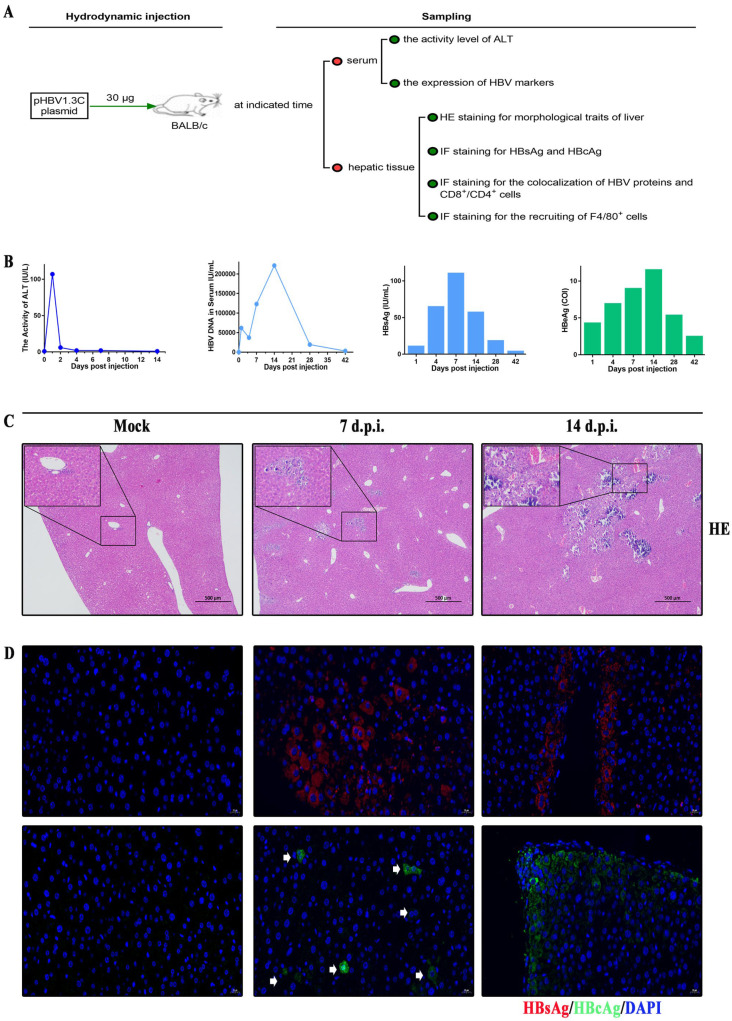
Viremia and histopathological traits of the BALB/c recipients injected with pHBV1.3C. (**A**) Schematic illustration of the BALB/c recipients injected with pHBV1.3C through hydrodynamic injection via the tail vein. The blood and hepatic tissue samples were collected at the indicated time for further analysis. (**B**) Viremia traits attributed to pHBV1.3C. Mouse serum data: ALT activity in the serum was measured by kinetic analysis at indicated time points. HBV DNA quantification in the serum was performed by qPCR. HBsAg and HBeAg levels were measured using ECL assays. (**C**) Histological analysis of the cross-sections was performed with HE staining (magnification ×40, scale bar 500 μm). (**D**) IF staining was conducted to evaluate the hepatic expression of HBsAg (red) and HBcAg (green) (magnification ×400, scale bar 20 μm). The white arrows pointed out the HBcAg^+^ hepatocytes. ALT, alanine transaminase; HE, hematoxylin–eosin; IF, immunofluorescence; Mock, negative control, pcDNA3.1(+) injection at one d.p.i.; d.p.i., days post-hydrodynamic injection; HBsAg, hepatitis B surface antigen; HBeAg, hepatitis B e antigen; HBcAg, hepatitis B core antigen.

Next, we further investigated the virological traits induced by pHBV1.3C in vivo. The BALB/c recipients were injected with the plasmids by HDI via the tail vein and sampled as described in Figure 3A. The kinetic analysis revealed that the activity of ALT peaked at one day post-HDI (d.p.i.) and then declined to the baseline level (≤50 IU/L) (Figure 3B). The serological data showed that the circulatory HBV DNA peaked at 14 d.p.i. and was still detectable at 42 d.p.i. (Figure 3B). Further ECL assays demonstrated that the serum level of HBsAg increased promptly within seven d.p.i. but decreased thereafter (Figure 3B). HBeAg gradually accumulated within 14 d.p.i. and remained positive until 42 d.p.i. (Figure 3B). These results together suggested that pHBV1.3C was successfully delivered into the murine liver through hydrodynamic injection and that it induced HBV viremia in the murine recipients.

### 3.4. Histopathological Characteristics in BALB/c Recipients Injected with pHBV1.3C

We also examined the histopathological effects in the murine hepatic tissue owing to the injection of pHBV1.3C. As expected, the HE staining results showed that pHBV1.3C triggered inflammatory infiltration and induced focal or piecemeal necrosis in the hepatic tissues of different BALB/c recipients as indicated in Figure 3C, in contrast to the mock-treated control. These findings indicated that pHBV1.3C resulted in different degrees of morphological changes in livers. Subsequently, the hepatic tissues were subjected to the IF staining to determine the expression of HBV proteins. The IF staining confirmed that the cytoplasmic HBsAg and HBcAg were positive in the recipients’ hepatocytes (Figure 3D). Taken together, our data confirmed that pHBV1.3C was able to replicate and express HBV proteins in the host hepatic cells and induce histopathological injury in vivo.

### 3.5. Hepatic Immunopathological Characteristics in BALB/c Recipients Injected with pHBV1.3C

**Figure 4:**
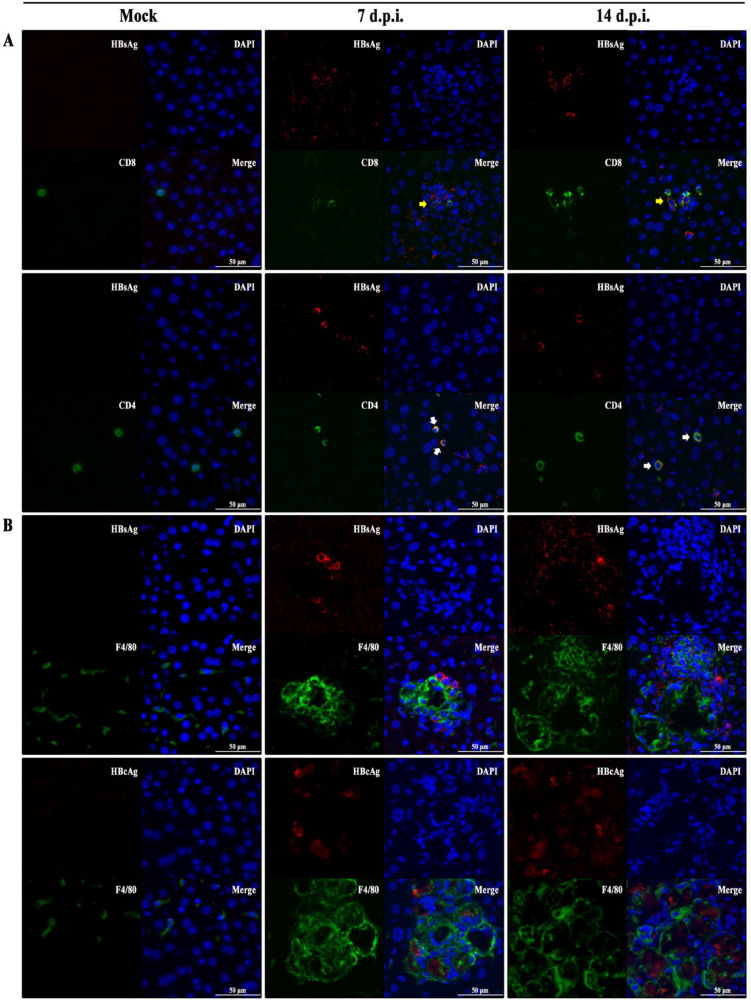
Immunological response of BALB/c recipients injected with pHBV1.3C. (**A**) IF staining was conducted to examine the colocalization of HBsAg (red) and CD8^+^ or CD4^+^ T (green) cells. HBsAg-specific CD8^+^ T cells were marked with a yellow arrow in the infection focus, and HBsAg-specific CD4^+^ T cells were marked with a white arrow in the livers. (**B**) IF staining revealed that F8/40^+^ cells (green) were apparently recruited around HBsAg (red)–positive cells or the HBcAg (red)–positive nidus in the livers. IF, immunofluorescence; Mock, negative control, pcDNA3.1(+) injection at one d.p.i.; d.p.i., days post hydrodynamic injection; HBsAg, hepatitis B surface antigen; HBcAg, hepatitis B core antigen; magnification ×400, scale bar 50 μm.

Given that the virus-specific immune response is critical for the host against HBV in vivo, we investigated three typical markers of the adaptive and innate immune cells within the liver of the recipients. Then, IF staining of CD8, CD4, and F4/80 was performed to detect CD8^+^ T cells, CD4^+^ T cells, and mouse liver-resident macrophages [32], respectively. The IF assays revealed the colocalization of HBsAg with CD8^+^ or CD4^+^ T cells in the livers of pHBV1.3C-injected recipients (Figure 4A). The staining results also indicated the abundant recruitment of F4/80^+^ cells around HBV-related necrotic lesions in the pHBV1.3C-injected livers (Figure 4B), which may provide a hint for the mechanism of pHBV1.3C-induced necrosis.

## 4. Discussion

HBV genotype C is still mostly responsible for the prevalence of HBV infections in China [16]. The replication of HBV is catalyzed by HBV DNA polymerase that lacks a proofreading ability, which explains why its mutation rate is 10 times higher than that of other DNA viruses. The high mutation rate allows HBV to acclimatize to the host environment but also poses an obvious burden for the elimination of HBV [33,34]. Thus, the consensus sequence of HBV genotype C could pave the way for an intensive and robust understanding of the virus. Moreover, scientific validation is required to develop a convenient infectious clone replicated in vitro and in vivo.

First, we established the representative consensus sequence of HBV genotype C, using 138 full-length genomes randomly obtained from the Chinese population. The entropy analysis of the 138 genotype C genomes showed that the polymorphism in the nonoverlapping regions was more frequent than that in the overlapping regions, which may suggest the constrained complexity distribution of those sequences [35]. Phylogenetic tree analysis showed that the 138 full-length genomes belong to HBV genotype C and are distinct from other HBV genotypes. The subgenotype with the highest proportion among the 138 genotype C genomes was C2, which is dominant in China [19]. The pcDNA3.1(+) vector containing the HBV genome is commonly used to study the virological and immunological traits of HBV under the control of CMV promoter/enhancer [25,36,37]. Different lengths of the HBV genome inserted into the plasmid backbone affect the expression level of HBV biomarkers [25]. Other HBV-related studies have shown that the 1.3-fold HBV genome plasmid ensures the effective transcription of 3.5 kb pgRNA [28,38,39]. The 1.3-fold consensus sequence contains HBV promoter elements and enhancer elements (Supplementary Materials Figure S1), which modulate the expression of HBV genes. Hence, we constructed the infectious plasmid with a 1.3-fold full-length consensus sequence, namely, pHBV1.3C, for further validation.

Second, pHBV1.3C was practically validated via its in vitro performances. Other studies have shown that human hepatoma cell lines, i.e., HepG2 cells and Huh7 cells, provide robust systems to study the replication of HBV [31,40]. Once the plasmids are delivered into the hepatoma cell lines, the host’s RNA polymerase with cell-specific transcription factors uses the HBV genome template for all viral transcripts in the nucleus. HBV ORFs are encoded into genomic and subgenomic transcripts. Genomic transcripts, acting as mRNAs for encoding HBeAg, HBcAg, and polymerase, are referred to as HBV pgRNA. The pgRNA is reverse-transcribed to HBV DNA. The subgenomic transcripts act as templates for HBsAg and HBx [4]. Our in vitro results revealed that the extracellular HBV DNA levels of pHBV1.3C-transfected HepG2 cells and Huh7 cells were significantly higher than those of pHBV1.3B, which correlate with the more active replication of HBV genotype C in patients [35]. The qPCR results also revealed that HBV pgRNA—the indicator of the HBV replication in the host cells—was detectable in the pHBV1.3C-transfected cells. The in vitro replicative capacity was further verified through the expression of HBV proteins. The IF staining revealed the cytoplasmic colocalization of HBsAg and HBcAg in transfected cells at 96 h. In keeping with the life cycle of HBV in HepG2 cells and Huh7 cells, HBsAg and HBeAg apparently accumulate in a time-dependent manner, which further supports the replication and expression of pHBV1.3C in vitro.

Subsequently, the in vivo investigations showed HBV viremia and histopathological findings of the murine recipients induced by pHBV1.3C. Hydrodynamic injection via the tail vein is an efficient method to deliver the genetic material into the murine liver. It involves a rapid liquid injection of an appropriate volume with HBV plasmids, which results in viremia, histopathological traits, and even immune response [38,41]. The outcome of hydrodynamic injection with HBV plasmids depends on the host immune response, as it is during a natural infection [38]. BALB/c mice and C57BL/6 mice, which are immunocompetent, are widely used to study acute or chronic HBV infection in vivo via HDI, respectively [31,42,43,44]. The course of acute HBV infection is characterized by an initial peak in HBV DNA and HBV proteins, such as HBsAg and HBeAg [45]. HBV proteins and their respective antibodies are detectable at seven weeks in BALB/c mice [42], and HBV intermediates and proteins are detectable for up to one year in C57BL/6 mice [43]. C57BL/6 mice—susceptible to chronic HBV infection—may yield positive HBV-related antibodies [44] and intrahepatic covalently closed circular DNA (cccDNA) [46]. In our study, we used BALB/c mice as the murine recipients. The kinetic assays indicated that the activity of serum ALT—a marker of liver damage—peaked at one d.p.i., which might result from the effective delivery of pHBV1.3C into the liver via hydrodynamic injection; however, the ALT declined to the baseline level thereafter. Normal ALT levels do not mean that the liver has no inflammation [47]. The serological profiles also proved that the pHBV1.3C induced HBV viremia with HBV DNA, HBsAg, and HBeAg serostatus (Supplementary Materials Table S3). These HBV markers could peak within two weeks upon the acute HBV infection and then decrease, which is probably related to the host’s antivirus immune response [48,49]. The absence or presence of anti-HBs, anti-HBc, and anti-HBe has been used to distinguish among different clinical phases of HBV infection [50]. The HBsAg is weakly immunogenic in the absence of adjuvants. Antonio [50] found that the anti-HBs appear after 10 weeks of HBV infection. HBeAg is a non-particulate protein of HBV. Seroconversion from HBeAg to anti-HBe is an important hallmark of disease progression, which is typically associated with a transition to an inactive infection with a low level of HBV DNA [9]. Spontaneous HBeAg seroconversion in patients with HBeAg-positive CHB is rare within six months [51]. Anti-HBc is used as a marker of ongoing or prior HBV contact. Other researchers have shown that the anti-HBc is produced after six weeks of acute infection [52]. The dynamics of these antibodies need to be further investigated in the pHBV1.3C-HDI mouse model. Furthermore, our histological analysis using HE staining showed that pHBV1.3C triggered hepatic damage (inflammatory infiltration and focal necrosis or piecemeal necrosis) within the BALB/c recipients. Consistent with the human hepatoma cells, the IF staining also confirmed the expression of HBsAg and HBcAg in the hepatocytes because of the injection of pHBV1.3C. Together with the in vitro study on the hepatoma cell lines, our in vivo investigations consistently supported the HBV replication and expression of pHBV1.3C.

HBV infection is not directly cytopathic to hepatocytes but rather through the immune system. The host’s innate and adaptive immune response promotes the clearance of HBV in patients with acute hepatitis B [52]. The inflammatory infiltration and hepatic necrosis might be associated with the complicated immune response in a bid to eliminate the HBV attack [53,54,55]. To determine the infiltration of immune cells in pHBV1.3C-induced acute liver injury, we studied the relationship of three typical immune cells with HBV proteins. HBV-specific CD8^+^ T cells, helped by CD4^+^ T cells, are crucial for the spontaneous resolution of acute HBV infection [56]. The peak appearance of HBV-specific CD8^+^ T cells is often concomitant with the peak of liver damage, but the quantity of HBV-specific CD8^+^ T cells is low [50]. Recent studies have shown that CD8^+^ T cells recognize various HBV epitopes with distinct phenotypic and functional features [57]. In this study, we observed the colocalization of HBsAg and CD8^+^ T cells or CD4^+^ T cells in the pHBV1.3C-injected livers. HBsAg-specific CD8^+^ T cells are one of the induced effectors of HBV clearance in the HBV infection model [58]. They are more responsive to metabolic intervention than HBcAg-specific T cells (undetectable in the infected liver within two weeks in this study) [57]. Limited amounts of viral antigen influence the cytokines production in HBV-specific CD8^+^ T cells [59]. Other studies have shown that HBV-specific CD8^+^ T cells indirectly cause liver damage by triggering the recruitment of inflammatory infiltration composed of macrophages [37,60]. The largest innate immune cell population in the liver is the tissue-resident macrophages, also known as Kupffer cells. F4/80 is a representative surface marker of mouse monocyte macrophages [61]. There may be two F8/40^+^ Kupffer cells subsets, with phagocytic activity and cytokine-producing capacity [62]. Our data revealed that F8/40^+^ cells were abundantly recruited around the HBsAg^+^ cells or HBcAg^+^ nidus, which may lead to the progression of liquefaction of necrotic tissues attributed to pHBV1.3C. Here, the inflammatory liver lesions may not be the result of the infiltration of CD8^+^ T cells, which target the infected hepatocytes through cytokines and lysis, but rather the result of Kupffer cells’ phagocytic activity. The molecular details remain to be fully investigated in the future.

Taken together, our aforementioned investigations provide a valuable tool to investigate HBV genotype C. The chemically synthesized consensus sequence of genotype C provides valuable genic background for the research of the virus life cycle, virus–host interaction, drug resistance, new agent screening, and treatment outcomes. Moreover, our previous study reported that some HBsAg mutations play a coordinated role in the pathogenesis of HBV [28]. The new infectious plasmids could be used to understand the underlying mechanism of single mutation and coordinated mutation among HBV genotype C, which would be beneficial to optimizing virus testing and developing corresponding strategies to eradicate HBV infection.

## 5. Conclusions

Notably, our data illustrates the feasible replication and expression of the synthesized consensus genome of HBV genotype C from the Chinese population. The in vitro results revealed that pHBV1.3C (including the 1.3-fold consensus genome of HBV genotype C) can produce the biomarkers of HBV in host HepG2 cells and Huh7 cells. Further in vivo investigations showed that pHBV1.3C induces HBV viremia and HBV histopathological traits and triggers immunopathological changes, thereby causing necroinflammation in the liver of BALB/c recipients. Our study provides a convenient tool to understand the pathogenesis and virus-related traits of HBV genotype C.

## Data Availability

The data are contained within the article and Supplementary Materials. Further inquiries can be directed to the corresponding author/s.

## Supplementary Materials

The following supporting information can be downloaded at https://www.mdpi.com/article/10.3390/v15122302/s1, Figure S1: The established consensus sequence of HBV genotype C; Table S1: The translated amino acid sequences of the four open reading frames (ORFs: P, preC/C, preS/S, X) from the consensus sequence of genotype C; Table S2: Positional frequency report of 138 HBV genotype C genomes; Table S3: Serum data of the mice recipients injected with pHBV1.3C.

## References

[1] World Health Organization (2017). Global Hepatitis Report 2017.

[2] Razavi H. (2020). Global Epidemiology of Viral Hepatitis. Gastroenterol. Clin. N. Am..

[3] El-Serag H.B. (2012). Epidemiology of Viral Hepatitis and Hepatocellular Carcinoma. Gastroenterology.

[4] Lamontagne R.J., Bagga S., Bouchard M.J. (2016). Hepatitis B virus molecular biology and pathogenesis. Hepatoma Res..

[5] Waheed Y., Siddiq M., Jamil Z., Najmi M.H. (2018). Hepatitis elimination by 2030: Progress and challenges. World J. Gastroenterol..

[6] Razavi-Shearer D., Gamkrelidze I., Nguyen M.H., Chen D.-S., Van Damme P., Abbas Z., Abdulla M., Abou Rached A., Adda D., Aho I. (2018). Global prevalence, treatment, and prevention of hepatitis B virus infection in 2016: A modelling study. Lancet Gastroenterol. Hepatol..

[7] Liu J., Liang W., Jing W., Liu M. (2019). Countdown to 2030: Eliminating hepatitis B disease, China. Bull. World Health Organ..

[8] Karayiannis P. (2017). Hepatitis B virus: Virology, molecular biology, life cycle and intrahepatic spread. Hepatol. Int..

[9] Coffin C.S., Zhou K., Terrault N.A. (2019). New and Old Biomarkers for Diagnosis and Management of Chronic Hepatitis B Virus Infection. Gastroenterology.

[10] Tong S., Revill P. (2016). Overview of viral replication and genetic variability. J. Hepatol..

[11] Hu J., Liu K. (2017). Complete and Incomplete Hepatitis B Virus Particles: Formation, Function, and Application. Viruses.

[12] Tatematsu K., Tanaka Y., Kurbanov F., Sugauchi F., Mano S., Maeshiro T., Nakayoshi T., Wakuta M., Miyakawa Y., Mizokami M. (2009). A genetic variant of hepatitis B virus divergent from known human and ape genotypes isolated from a Japanese patient and provisionally assigned to new genotype J. J. Virol..

[13] Wang Y., Shan X., Liang Z., Shan Y., Huang W., Zhang D., Zen A., Zhou X., Zhao Y., Gong X. (2015). Deep sequencing analysis of HBV genotype shift and correlation with antiviral efficiency during adefovir dipivoxil therapy. PLoS ONE.

[14] Araujo N.M., Teles S.A., Spitz N. (2020). Comprehensive Analysis of Clinically Significant Hepatitis B Virus Mutations in Relation to Genotype, Subgenotype and Geographic Region. Front. Microbiol..

[15] Rajoriya N., Combet C., Zoulim F., Janssen H.L.A. (2017). How viral genetic variants and genotypes influence disease and treatment outcome of chronic hepatitis B. Time for an individualised approach?. J. Hepatol..

[16] Li H.M., Wang J.Q., Wang R., Zhao Q., Li L., Zhang J.P., Shen T. (2015). Hepatitis B virus genotypes and genome characteristics in China. World J. Gastroenterol..

[17] Tian Q., Jia J. (2016). Hepatitis B virus genotypes: Epidemiological and clinical relevance in Asia. Hepatol. Int..

[18] Norder H., Courouce A.M., Coursaget P., Echevarria J.M., Lee S.D., Mushahwar I.K., Robertson B.H., Locarnini S., Magnius L.O. (2004). Genetic diversity of hepatitis B virus strains derived worldwide: Genotypes, subgenotypes, and HBsAg subtypes. Intervirology.

[19] Bello K.E., Jusoh T.N.A.M., Irekeola A.A., Abu N., Amin N.A.Z.M., Mustaffa N., Shueb R.H. (2023). A Recent Prevalence of Hepatitis B Virus (HBV) Genotypes and Subtypes in Asia: A Systematic Review and Meta-Analysis. Healthcare.

[20] Feng Y., Ran J., Feng Y.-M., Miao J., Zhao Y., Jia Y., Li Z., Yue W., Xia X. (2020). Genetic diversity of hepatitis B virus in Yunnan, China: Identification of novel subgenotype C17, an intergenotypic B/I recombinant, and B/C recombinants. J. Gen. Virol..

[21] Kramvis A. (2014). Genotypes and genetic variability of hepatitis B virus. Intervirology.

[22] Orito E. (2001). A case-control study for clinical and molecular biological differences between hepatitis B viruses of genotypes B and C. Hepatology.

[23] Chan H.L., Hui A.Y., Wong M.L., Tse A.M., Hung L.C., Wong V.W., Sung J.J. (2004). Genotype C hepatitis B virus infection is associated with an increased risk of hepatocellular carcinoma. Gut.

[24] Sunbul M. (2014). Hepatitis B virus genotypes: Global distribution and clinical importance. World J. Gastroenterol..

[25] Li X., Liu G., Chen M., Yang Y., Xie Y., Kong X. (2016). A Novel Hydrodynamic Injection Mouse Model of HBV Genotype C for the Study of HBV Biology and the Anti-Viral Activity of Lamivudine. Hepat. Mon..

[26] Chen Q.Y., Harrison T.J., Sabin C.A., Li G.J., Huang G.M., Yang J.Y., Wang X.Y., Li H., Liu M.H., Fang Z.L. (2014). The Effect of HBV Genotype C on the Development of HCC Differs between Wild-Type Viruses and Those with BCP Double Mutations (T(1762)A(1764)). Hepat. Mon..

[27] Liu T., Liu A., Liu Y., Cen S., Zhang Q. (2022). In vitro investigation of HBV clinical isolates from Chinese patients reveals that genotype C isolates possess higher infectivity than genotype B isolates. Virol. Sin..

[28] Wang H., Wang M., Huang J., Xu R., Liao Q., Shan Z., Zheng Y., Rong X., Tang X., Li T. (2020). Novel hepatitis B virus surface antigen mutations associated with occult genotype B hepatitis B virus infection affect HBsAg detection. J. Viral Hepat..

[29] Wang H. (2021). Molecular Biological Characteristics of Hepatitis B Virus S Gene and Its Effect on the Expression of Hepatitis B Surface Antigen in Blood Donors with Occult Hepatitis B Virus Infection in Guangzhou Doctoral Dissertation 2020. Ph.D. Thesis.

[30] Hall B.G. (2013). Building phylogenetic trees from molecular data with MEGA. Mol. Biol. Evol..

[31] Hu J., Lin Y.Y., Chen P.J., Watashi K., Wakita T. (2019). Cell and Animal Models for Studying Hepatitis B Virus Infection and Drug Development. Gastroenterology.

[32] Austyn J., Gordon S. (1981). F4/80, a monoclonal antibody directed specifically against the mouse macrophage. Eur. J. Immunol..

[33] Pawlotsky J.M. (2005). The concept of hepatitis B virus mutant escape. J. Clin. Virol..

[34] Valaydon Z.S., Locarnini S.A. (2017). The Virological Aspects of Hepatitis B. Best Pract. Res. Clin. Gastroenterol..

[35] Liu F., Yu D.M., Huang S.Y., Yu J.L., Zhang D.H., Gong Q.M., Zhang X.X. (2014). Clinical Implications of Evolutionary Patterns of Homologous, Full-Length Hepatitis B Virus Quasispecies in Different Hosts after Perinatal Infection. J. Clin. Microbiol..

[36] Wang H., Liao F., Xie J., Gao W., Wang M., Huang J., Xu R., Liao Q., Shan Z., Zheng Y. (2021). E2 Site Mutations in S Protein Strongly Affect Hepatitis B Surface Antigen Detection in the Occult Hepatitis B Virus. Front. Microbiol..

[37] Sitia G., Isogawa M., Iannacone M., Campbell I.L., Chisari F.V., Guidotti L.G. (2004). MMPs are required for recruitment of antigen-nonspecific mononuclear cells into the liver by CTLs. J. Clin. Investig..

[38] Yang P.L., Althage A., Chung J., Chisari F.V. (2002). Hydrodynamic injection of viral DNA: A mouse model of acute hepatitis B virus infection. Proc. Natl. Acad. Sci. USA.

[39] Zhang Z., Xia J., Sun B., Dai Y., Li X., Schlaak J.F., Lu M. (2015). In vitro and in vivo replication of a chemically synthesized consensus genome of hepatitis B virus genotype B. J. Virol. Methods.

[40] Sun D., Nassal M. (2006). Stable HepG2- and Huh7-based human hepatoma cell lines for efficient regulated expression of infectious hepatitis B virus. J. Hepatol..

[41] Dandri M., Petersen J. (2017). Animal models of HBV infection. Best Pr. Res. Clin. Gastroenterol..

[42] Chen J., Liu B., Tang X., Zheng X., Lu J., Zhang L., Wang W., Candotti D., Fu Y., Allain J.-P. (2021). Role of core protein mutations in the development of occult HBV infection. J. Hepatol..

[43] Huang L.-R., Wu H.-L., Chen P.-J., Chen D.-S. (2006). An immunocompetent mouse model for the tolerance of human chronic hepatitis B virus infection. Proc. Natl. Acad. Sci. USA.

[44] Yang D., Liu L., Zhu D., Peng H., Su L., Fu Y.-X., Zhang L. (2013). A mouse model for HBV immunotolerance and immunotherapy. Cell. Mol. Immunol..

[45] Suresh M., Czerwinski S., Murreddu M.G., Kallakury B.V., Ramesh A., Gudima S.O. (2019). Innate and adaptive immunity associated with resolution of acute woodchuck hepatitis virus infection in adult woodchucks. PloS Pathog..

[46] Wang L., Cao M., Wei Q.L., Zhao Z.H., Xiang Q., Wang H.J., Zhang H.T., Lai G.Q. (2017). A new model mimicking persistent HBV e antigen-negative infection using covalently closed circular DNA in immunocompetent mice. PLoS ONE.

[47] Chang M.H., Hwang L.-Y., Hsu H.C., Lee C.Y., Beasley R.P. (1988). Prospective study of asymptomatic HBsAg carrier children infected in the perinatal period clinical and liver histologic studies. Hepatology.

[48] Dusseaux M., Masse-Ranson G., Darche S., Ahodantin J., Li Y., Fiquet O., Beaumont E., Moreau P., Riviere L., Neuveut C. (2017). Viral Load Affects the Immune Response to HBV in Mice with Humanized Immune System and Liver. Gastroenterology.

[49] Shiina M., Yamada N., Sugiyama R., Murayama A., Aly H.H., Muramatsu M., Wakita T., Imawari M., Kato T. (2019). Hepatitis B Virus Genotype-Dependent Vulnerability of Infected Cells to Immune Reaction in the Early Phase of Infection. Front. Microbiol..

[50] Bertoletti A., Ferrari C. (2016). Adaptive immunity in HBV infection. J. Hepatol..

[51] Song B.-C., Cho Y.-K., Jwa H., Choi E.K., Kim H.U., Song H.J., Na S.-Y., Boo S.-J., Jeong S.U. (2014). Is it necessary to delay antiviral therapy for 3-6 months to anticipate HBeAg seroconversion in patients with HBeAg-positive chronic hepatitis B in endemic areas of HBV genotype C?. Clin. Mol. Hepatol..

[52] Zheng J.R., Wang Z.L., Feng B. (2022). Hepatitis B functional cure and immune response. Front. Immunol..

[53] Krebs K., Bottinger N., Huang L.R., Chmielewski M., Arzberger S., Gasteiger G., Jager C., Schmitt E., Bohne F., Aichler M. (2013). T cells expressing a chimeric antigen receptor that binds hepatitis B virus envelope proteins control virus replication in mice. Gastroenterology.

[54] Kah J., Koh S., Volz T., Ceccarello E., Allweiss L., Lutgehetmann M., Bertoletti A., Dandri M. (2017). Lymphocytes transiently expressing virus-specific T cell receptors reduce hepatitis B virus infection. J. Clin. Investig..

[55] Xie Y., Zhong K.B., Hu Y., Xi Y.L., Guan S.X., Xu M., Lin Y., Liu F.Y., Zhou W.J., Gao Y. (2022). Liver infiltration of multiple immune cells during the process of acute liver injury and repair. World J. Gastroenterol..

[56] Shin E.C., Sung P.S., Park S.H. (2016). Immune responses and immunopathology in acute and chronic viral hepatitis. Nat. Rev. Immunol..

[57] Fu Y.L., Zhou S.N., Hu W., Li J., Zhou M.J., Li X.Y., Wang Y.Y., Zhang P., Chen S.Y., Fan X. (2023). Metabolic interventions improve HBV envelope-specific T-cell responses in patients with chronic hepatitis B. Hepatol. Int..

[58] Yang P.L., Althage A., Chung J., Maier H., Wieland S., Isogawa M., Chisari F.V. (2010). Immune effectors required for hepatitis B virus clearance. Proc. Natl. Acad. Sci. USA.

[59] Gehring A.J., Sun D., Kennedy P.T.F., Hoen E.N., Lim S.G., Wasser S., Selden C., Maini M.K., Davis D.M., Nassal M. (2007). The level of viral antigen presented by hepatocytes influences CD8 T-cell function. J. Virol..

[60] Bertoletti A., Maini M.K., Ferrari C. (2010). The host-pathogen interaction during HBV infection: Immunological controversies. Antivir. Ther..

[61] Boltjes A., Movita D., Boonstra A., Woltman A.M. (2014). The role of Kupffer cells in hepatitis B and hepatitis C virus infections. J. Hepatol..

[62] Kinoshita M., Uchida T., Sato A., Nakashima M., Nakashima H., Shono S., Habu Y., Miyazaki H., Hiroi S., Seki S. (2010). Characterization of two F4/80-positive Kupffer cell subsets by their function and phenotype in mice. J. Hepatol..

