# Supplementary material for: Replication and Expression of the Consensus Genome of Hepatitis B Virus Genotype C from the Chinese Population
Source: Viruses. 2023 Nov 23;15(12):2302. doi: 10.3390/v15122302 (PMC10747539; doi:10.3390/v15122302)
Supplement: Supplementary file 1 [file viruses-15-02302-s001.zip › viruses-2530543-supplementary.pdf]

Supplementary Figure

Supplementary Figure S1

```
                  10        20        30        40        50        60        70        80        90       100
001-100   ....|....|....|....|....|....|....|....|....|....|....|....|....|....|....|....|....|....|....|....|
Ccs.TXT   CTCCACAACATTCCACCAAGCTCTGCTAGATCCCAGAGTGAGGGGCCTATACTTTCCTGCTGGTGGCTCCAGTTCCGGAACAGTAAACCCTGTTCCGACT

                 110       120       130       140       150       160       170       180       190       200
101-200   ....|....|....|....|....|....|....|....|....|....|....|....|....|....|....|....|....|....|....|....|
Ccs.TXT   ACTGCCTCTCCCATATCGTCAATCTTCTCGAGGACTGGGGACCCTGCACCGAACATGGAGAACACAACATCAGGATTCCTAGGACCCCTGCTCGTGTTAC
                                                                 S

                 210       220       230       240       250       260       270       280       290       300
201-300   ....|....|....|....|....|....|....|....|....|....|....|....|....|....|....|....|....|....|....|....|
Ccs.TXT   AGGCGGGGTTTTTCTTGTTGACAAGAATCCTCACAATACCACAGAGTCTAGACTCGTGGTGGACTTCTCTCAATTTTCTAGGGGGAGCACCCACGTGTCC

                 310       320       330       340       350       360       370       380       390       400
301-400   ....|....|....|....|....|....|....|....|....|....|....|....|....|....|....|....|....|....|....|....|
Ccs.TXT   TGGCCAAAATTCGCAGTCCCCAACCTCCAATCACTCACCAACCTCTTGTCCTCCAATTTGTCCTGGCTATCGCTGGATGTGTCTGCGGCGTTTTATCATA

                 410       420       430       440       450       460       470       480       490       500
401-500   ....|....|....|....|....|....|....|....|....|....|....|....|....|....|....|....|....|....|....|....|
Ccs.TXT   TTCCTCTTCATCCTGCTGCTATGCCTCATCTTCTTGTTGGTTCTTCTGGACTACCAAGGTATGTTGCCCGTTTGTCCTCTACTTCCAGGAACATCAACTA

                 510       520       530       540       550       560       570       580       590       600
501-600   ....|....|....|....|....|....|....|....|....|....|....|....|....|....|....|....|....|....|....|....|
Ccs.TXT   CCAGCACGGGACCATGCAAGACCTGCACGATTCCTGCTCAAGGAACCTCTATGTTTCCCTCTTGTTGCTGTACAAAACCTTCGGACGGAAATTGCACTTG

                 610       620       630       640       650       660       670       680       690       700
601-700   ....|....|....|....|....|....|....|....|....|....|....|....|....|....|....|....|....|....|....|....|
Ccs.TXT   TATTCCCATCCCATCATCCTGGGCTTTCGCAAGATTCCTATGGGAGTGGGCCTCAGTCCGTTTCTCCTGGCTCAGTTTACTAGTGCCATTTGTTCAGTGG

                 710       720       730       740       750       760       770       780       790       800
701-800   ....|....|....|....|....|....|....|....|....|....|....|....|....|....|....|....|....|....|....|....|
Ccs.TXT   TTCGTAGGGCTTTCCCCCACTGTTTGGCTTTCAGTTATATGGATGATGTGGTATTGGGGGCCAAGTCTGTACAACATCTTGAGTCCCTTTTTACCTCTAT

                 810       820       830       840       850       860       870       880       890       900
801-900   ....|....|....|....|....|....|....|....|....|....|....|....|....|....|....|....|....|....|....|....|
Ccs.TXT   TACCAATTTTCTTTTGTCTTTGGGTATACATTTGAACCCTAATAAAACCAAACGTTGGGGCTACTCCCTTAACTTCATGGGATATGTAATTGGAAGTTGG
                                           S

                 910       920       930       940       950       960       970       980       990      1000
901-1000  ....|....|....|....|....|....|....|....|....|....|....|....|....|....|....|....|....|....|....|....|
Ccs.TXT   GGTACTTTACCACAGGAACATATTGTACAAAAAATCAAGCAATGTTTTCGAAAACTGCCTGTAAATAGACCTATTGATTGGAAAGTATGTCAAAGAATTG
                                                                   Enhancer I
                1010      1020      1030      1040      1050      1060      1070      1080      1090      1100
1001-1100 ....|....|....|....|....|....|....|....|....|....|....|....|....|....|....|....|....|....|....|....|
Ccs.TXT   TGGGTCTTTTGGGCTTTGCTGCCCCTTTTACACAATGTGGCTATCCTGCCTTAATGCCTTTATATGCATGTATACAATCTAAGCAGGCTTTCACTTTCTC

                1110      1120      1130      1140      1150      1160      1170      1180      1190      1200
1101-1200 ....|....|....|....|....|....|....|....|....|....|....|....|....|....|....|....|....|....|....|....|
Ccs.TXT   GCCAACTTACAAGGCCTTTCTGTGTAAACAATATCTGAACCTTTACCCCGTTGCCCGGCAACGGTCAGGTCTCTGCCAAGTGTTTGCTGACGCAACCCCC

                1210      1220      1230      1240      1250      1260      1270      1280      1290      1300
1201-1300 ....|....|....|....|....|....|....|....|....|....|....|....|....|....|....|....|....|....|....|....|
Ccs.TXT   ACTGGATGGGGCTTGGCCATAGGCCATCAGCGCATGCGTGGAACCTTTGTGGCTCCTCTGCCGATCCATACTGCGGAACTCCTAGCAGCTTGTTTTGCTC

                1310      1320      1330      1340      1350      1360      1370      1380      1390      1400
1301-1400 ....|....|....|....|....|....|....|....|....|....|....|....|....|....|....|....|....|....|....|....|
Ccs.TXT   GCAGCCGGTCTGGAGCGAAACTTATCGGAACCGACAACTCTGTTGTCCTCTCTCGGAAATACACCTCCTTTCCATGGCTGCTAGGGTGTGCTGCCAACTG
                                                                                    X
                1410      1420      1430      1440      1450      1460      1470      1480      1490      1500
1401-1500 ....|....|....|....|....|....|....|....|....|....|....|....|....|....|....|....|....|....|....|....|
Ccs.TXT   GATCCTGCGCGGGACGTCCTTTGTCTACGTCCCGTCGGCGCTGAATCCCGCGGACGACCCGTCTCGGGGCCGTTTGGGACTCTACCGTCCCCTTCTTCGT

                1510      1520      1530      1540      1550      1560      1570      1580      1590      1600
1501-1600 ....|....|....|....|....|....|....|....|....|....|....|....|....|....|....|....|....|....|....|....|
Ccs.TXT   CTGCCGTTCCGGCCGACCACGGGGCGCACCTCTCTTTACGCGGTCTCCCCGTCTGTGCCTTCTCATCTGCCGGACCGTGTGCACTTCGCTTCACCTCTGC
                                                                        DR2
                1610      1620      1630      1640      1650      1660      1670      1680      1690      1700
1601-1700 ....|....|....|....|....|....|....|....|....|....|....|....|....|....|....|....|....|....|....|....|
Ccs.TXT   ACGTCGCATGGAGACCACCGTGAACGCCCACCAGGTCTTGCCCAAGGTCTTACATAAGAGGACTCTTGGACTCTCAGCAATGTCAACGACCGACCTTGAG
                                 P                                                                Enhancer II
                1710      1720      1730      1740      1750      1760      1770      1780      1790      1800
1701-1800 ....|....|....|....|....|....|....|....|....|....|....|....|....|....|....|....|....|....|....|....|
Ccs.TXT   GCATACTTCAAAGACTGTGTGTTTAAAGACTGGGAGGAGTTGGGGGAGGAGATTAGGTTAAAGGTCTTTGTACTAGGAGGCTGTAGGCATAAATTGGTCT

                1810      1820      1830      1840      1850      1860      1870      1880      1890      1900
1801-1900 ....|....|....|....|....|....|....|....|....|....|....|....|....|....|....|....|....|....|....|....|
Ccs.TXT   GTTCACCAGCACCATGCAACTTTTTCACCTCTGCCTAATCATCTCATGTTCATGTCCTACTGTTCAAGCCTCCAAGCTGTGCCTTGGGTGGCTTTGGGGC
                         Pre-C       DR1        X
                1910      1920      1930      1940      1950      1960      1970      1980      1990      2000
1901-2000 ....|....|....|....|....|....|....|....|....|....|....|....|....|....|....|....|....|....|....|....|
Ccs.TXT   ATGGACATTGACCCGTATAAAGAATTTGGAGCTTCTGTGGAGTTACTCTCTTTTTTGCCTTCTGACTTCTTTCCTTCTATTCGAGATCTCCTCGACACCG
          C                Pol-A
                2010      2020      2030      2040      2050      2060      2070      2080      2090      2100
2001-2100 ....|....|....|....|....|....|....|....|....|....|....|....|....|....|....|....|....|....|....|....|
Ccs.TXT   CCTCTGCTCTGTATCGGGAGGCCTTAGAGTCTCCGGAACATTGTTCACCTCACCATACAGCACTCAGGCAAGCTATTCTGTGTTGGGGTGAGTTGATGAA

                2110      2120      2130      2140      2150      2160      2170      2180      2190      2200
2101-2200 ....|....|....|....|....|....|....|....|....|....|....|....|....|....|....|....|....|....|....|....|
Ccs.TXT   TCTGGCCACCTGGGTGGGAAGTAATTTGGAAGACCCAGCATCCAGGGAATTAGTAGTCAGCTATGTCAATGTTAATATGGGCCTAAAAATCAGACAACTA

                2210      2220      2230      2240      2250      2260      2270      2280      2290      2300
```

```
2201-2300      ....|....|....|....|....|....|....|....|....|....|....|....|....|....|....|....|....|....|....|....|
Ccs.TXT        TTGTGGTTTCACATTTCCTGTCTTACTTTTGGAAGAGAAACTGTTCTTGAGTATTTGGTGTCTTTTGGAGTGTGGATTCGCACTCCTCCCGCTTACAGAC

                     2310      2320      2330      2340      2350      2360      2370      2380      2390      2400
2301-2400      ....|....|....|....|....|....|....|....|....|....|....|....|....|....|....|....|....|....|....|....|
Ccs.TXT        CACCAAATGCCCCTATCTTATCAACACTTCCGGAAACTACTGTTGTTAGACGACGAGGCAGGTCCCCTAGAAGAAGAACTCCCTCGCCTCGCAGACGAAG
                      P
                     2410      2420      2430      2440      2450      2460      2470      2480      2490      2500
2401-2500      ....|....|....|....|....|....|....|....|....|....|....|....|....|....|....|....|....|....|....|....|
Ccs.TXT        GTCTCAATCGCCGCGTCGCAGAAGATCTCAATCTCGGGAATCTCAATGTTAGTATCCCTTGGACTCATAAGGTGGGAAACTTTACTGGGCTTTATTCTTC
                                                                C
                     2510      2520      2530      2540      2550      2560      2570      2580      2590      2600
2501-2600      ....|....|....|....|....|....|....|....|....|....|....|....|....|....|....|....|....|....|....|....|
Ccs.TXT        TACTGTACCTGTCTTTAATCCTGAGTGGCAAACTCCCTCCTTTCCTCACATTCATTTACAGGAGGACATTATTAATAGATGTCAACAATATGTGGGCCCT

                     2610      2620      2630      2640      2650      2660      2670      2680      2690      2700
2601-2700      ....|....|....|....|....|....|....|....|....|....|....|....|....|....|....|....|....|....|....|....|
Ccs.TXT        CTTACAGTTAATGAAAAAAGGAGATTAAAATTAATTATGCCTGCTAGGTTCTATCCTAACCTTACCAAATATTTGCCCTTGGACAAAGGCATTAAACCTT

                     2710      2720      2730      2740      2750      2760      2770      2780      2790      2800
2701-2800      ....|....|....|....|....|....|....|....|....|....|....|....|....|....|....|....|....|....|....|....|
Ccs.TXT        ATTATCCTGAACATGCAGTTAATCATTACTTCAAAACTAGGCATTATTTACATACTCTGTGGAAGGCTGGCATTCTATATAAGAGAGAAACTACACGCAG

                     2810      2820      2830      2840      2850      2860      2870      2880      2890      2900
2801-2900      ....|....|....|....|....|....|....|....|....|....|....|....|....|....|....|....|....|....|....|....|
Ccs.TXT        CGCCTCATTTTGTGGGTCACCATATTCTTGGGAACAAGAGCTACAGCATGGGAGGTTGGTCTTCCAAACCTCGACAAGGCATGGGGACGAATCTTTCTGT
                                                             Pre-S1
                     2910      2920      2930      2940      2950      2960      2970      2980      2990      3000
2901-3000      ....|....|....|....|....|....|....|....|....|....|....|....|....|....|....|....|....|....|....|....|
Ccs.TXT        TCCCAATCCTCTGGGATTCTTTCCCGATCACCAGTTGGACCCTGCGTTCGGAGCCAACTCAAACAATCCAGATTGGGACTTCAACCCCAACAAGGATCAC

                     3010      3020      3030      3040      3050      3060      3070      3080      3090      3100
3001-3100      ....|....|....|....|....|....|....|....|....|....|....|....|....|....|....|....|....|....|....|....|
Ccs.TXT        TGGCCAGAGGCAAATCAGGTAGGAGCGGGAGCATTCGGGCCAGGGTTCACCCCACCACACGGCGGTCTTTTGGGGTGGAGCCCTCAGGCTCAGGGCATAT

                     3110      3120      3130      3140      3150      3160      3170      3180      3190      3200
3101-3200      ....|....|....|....|....|....|....|....|....|....|....|....|....|....|....|....|....|....|....|....|
Ccs.TXT        TGACAACAGTGCCAGCAGCACCTCCTCCTGCCTCCACCAATCGGCAGTCAGGAAGACAGCCTACTCCCATCTCTCCACCTCTAAGAGACAGTCATCCTCA
                      3210
3201-3215      ....|....|....|
Ccs.TXT        GGCCATGCAGTGGAA
                 Pre-S2
```

Figure S1. **The established consensus sequence of HBV genotype C.** The full-length consensus genome is composed of 3215 nucleotides. Start codon was marked with green. Stop codon was marked with red. DR1, DR2, Pol-A were marked with blue. Two enhancers were marked with orange. Four promoters (core promoter, CP, nt1613-1849; PreS1 promoter, SPⅠ, nt2718-2808; PreS2 promoter, SPⅡ, nt2983-3210; X promoter, XP, nt1171-1361).

Supplementary Tables

Supplementary Table S1. The translated amino acid sequences of the four open reading frames (*P*, *preS/S*, *preCore/Core*, *X*) from the consensus sequence of genotype C.

Title	Gene locus	Amino Acid sequences
Polymerase<br>(843 aa)	nt(2307..3215,1..1623)	1MPLSYQHFRK LLLLDDEAGP LEEELPRLAD EGLNRRVAED LNLGNLNVSI PWTHKVGNFT<br>61 GLYSSTVPVF NPEWQTPSFP HIHLQEDIIN RCQQYVGPLT VNEKRRLKLI MPARFYPNLT<br>121 KYLPLDKGIK PYYPEHAVNH YFKTRHYLHT LWKAGILYKR ETTRSASFCG SPYSWEQELQ<br>181 HGRLVFQTST RHGDESFCSQ SSGILSRSPV GPCVRSQLKQ SRLGLQPQQG SLARGKSGRS<br>241 GSIRARVHPT TRRSFGVEPS GSGHIDNSAS STSSCLHQSA VRKTAYSHLS TSKRQSSSGH<br>301 AVELHNIPPS SARSQSEGPI LSCWWLQFRN SKPCSDYCLS HIVNLLEDWG PCTEHGEHNI<br>361 RIPRTPARVT GGVFLVDKNP HNTTESRLVV DFSQFSRGST HVSWPKFAVP NLQSLTNLLS<br>421SNLSWLSLDV SAAFYHIPLH PAAMPHLLVG SSGLPRYVAR LSSTSRNINY QHGTMQDLHD<br>481 SCSRNLYVSL LLLYKTFGRK LHLYSHPIIL GFRKIPMGVG LSPFLLAQFT SAICSVVRRA<br>541 FPHCLAFSYM DDVVLGAKSV QHLESLFTSI TNFLLSLGIH LNPNKTKRWG YSLNFMGYVI<br>601 GSWGTLPQEH IVQKIKQCFR KLPVNRPIDW KVCQRIVGLL GFAAPFTQCG YPALMPLYAC<br>661 IQSKQAFTFS PTYKAFLCKQ YLNLYPVARQ RSGLCQVFAD ATPTGWGLAI GHQRMRGTFV<br>721 APLPIHTAEL LAACFARSRS GAKLIGTDNS VVLSRKYTSF PWLLGCAANW ILRGTSFVYV<br>781 PSALNPADDP SRGRLGLYRP LLRLPFRPTT GRTSLYAVSP SVPSHLPDRV HFASPLHVAW<br>841 RPP

Large S protein (400 aa)	nt(2848..3215,1..835)	1MGGWSSKPRQ GMGTNLSVPN PLGFFPDHQL DPAFGANSNN PDWDFNPNKD HWPEANQVGA<br>61 GAFGPGFTPP HGGLLGWSPQ AQGILTTVPA APPPASTNRQ SGRQPTPISP PLRDSHPQAM<br>121 QWNSTTFHQA LLDPRVRGLY FPAGGSSSGT VNPVPTTASP ISSIFSRTGD PAPNMENTTS<br>181 GFLGPLLVLQ AGFFLLTRIL TIPQSLDSWW TSLNFLGGAP TCPGQNSQSP TSNHSPTSCP<br>241 PICPGYRWMC LRRFIIFLFI LLLCLIFLLV LLDYQGMLPV CPLLPGTSTT STGPCKTCTI<br>301 PAQGTSMFPS CCCTKPSDGN CTCIPIPSSW AFARFLWEWA SVRFSWLSLL VPFVQWFVGL<br>361 SPTVWLSVIW MMWYWGPSLY NILSPFLPLL PIFFCLWVYI
middle S protein (281 aa)	nt(3205..3215,1..835)	1 MQWNSTTFHQ ALLDPRVRGL YFPAGGSSSG TVNPVPTTAS PISSIFSRTG DPAPNMENTT<br>61 SGFLGPLLVL QAGFFLLTRI LTIPQSLDSW WTSLNFLGGA PTCPGQNSQS PTSNHSPTSC<br>121 PPICPGYRWM CLRRFIIFLF ILLLCLIFLL VLLDYQGMLP VCPLLPGTST TSTGPCKTCT<br>181 IPAQGTSMFP SCCCTKPSDG NCTCIPIPSS WAFARFLWEW ASVRFSWLSL LVPFVQWFVG<br>241 LSPTVWLSVI WMMWYWGPSL YNILSPFLPL LPIFFCLWVY I
S protein (226 aa)	nt(155..835)	1 MENTTSGFLG PLLVLQAGFF LLTRILTIPQ SLDSWWTSLN FLGGAPTCPG QNSQSPTSNH<br>61 SPTSCPPICP GYRWMCLRRF IIFLFILLLC LIFLLVLLDY QGMLPVCPLL PGTSTTSTGP<br>121 CKTCTIPAQG TSMFPSCCCT KPSDGNCTCI PIPSSWAFAR FLWEWASVRF SWLSLLVPFV<br>181 QWFVGLSPTV WLSVIWMMWY WGPSLYNILS PFLPLLPIFF CLWVYI
X protein (154 aa)	nt(1374..1838)	1 MAARVCCQLD PARDVLCLRP VGAESRGRPV SGPFGTLPSP SSSAVPADHG AHLSLRGLPV<br>61 CAFSSAGPCA LRFTSARRME TTVNAHQVLP KVLHKRTLGL SAMSTTDLEA YFKDCVFKDW<br>121 EELGEEIRLK VFVLGGCRHK LVCSPAPCNF FTSA
precore/core protein (212 aa)	nt(1814..2452)	1 MQLFHLCLII SCSCPTVQAS KLCLGWLWGM DIDPYKEFGA SVELLSFLPS DFFPSIRDLL<br>61 DTASALYREA LESPEHCSPH HTALRQAILC WGELMNLATW VGSNLEDPAS RELVVSYVNV<br>121 NMGLKIRQLL WFHISCLTFG RETVLEYLVS FGVWIRTPPA YRPPNAPILS TLPETTVVRR<br>181 RGRSPRRRTP SPRRRRSQSP RRRRSQSRES QC
Core protein (183 aa)	nt(1901..2452)	1 MDIDPYKEFG ASVELLSFLP SDFFPSIRDL LDTASALYRE ALESPEHCSP HHTALRQAIL<br>61 CWGELMNLAT WVGSNLEDPA SRELVVSYVN VNMGLKIRQL LWFHISCLTF GRETVLEYLV<br>121 SFGVWIRTPP AYRPPNAPIL STLPETTVVR RRGRSPRRRT PSPRRRRSQS PRRRRSQSRE<br>181 SQC

Supplementary Table 2

Positional frequency report of 138 HBV genotype C genomes.

The detailed data were provided in Excel.

Supplementary Table S3. Serum data of the mice recipients injected with pHBV1.3C.

Marker	BALB/c	Different kinetics of serum markers at the indicated days with pHBV1.3C hydrodynamic injection					
(Unit)	mouse	1	4	7	14	28	42
HBsAg (IU/mL)	#1	12.78	173.95	190.00	1.24	*	*
	#2	11.67	85.83	131.17	0.85	0.15	*
	#3	4.65	39.40	68.36	0.14	/	/
	#4	11.72	65.56	111.10	58.08	19.18	4.7
	#5	3.98	40.61	86.23	/	/	/
	#6	21.32	/	/	/	/	/
HBeAg (COI)	#1	10.67	37.62	25.13	0.31	0.31	0.29
	#2	5.15	10.21	14.55	3.80	0.88	0.13
	#3	2.74	4.97	4.62	0.59	/	/
	#4	4.37	7.00	9.06	11.61	5.44	2.57
	#5	4.25	3.63	6.36	/	/	/
	#6	2.67	/	/	/	/	
ALT (IU/mL)	#1	694	2	3	2	1	1
	#2	107	2	1	1	2	3
	#3	160	6	3	2	/	/
	#4	107	2	2	1	1	2
	#5	181	5	4	/	/	/
	#6	65	/	/	/	/	/
HBV DNA (IU/mL)	#1	211274.63	17900.70	43779.01	*N*	*N*	*N*
	#2	827022.20	44660.18	714040.90	1026.92	*N*	*N*
	#3	602078.80	42710.64	102043.20	24852.68	/	/
	#4	61631.79	37125.55	122997.11	221303.11	19422.89	2958.24
	#5	2718457.00	28256.99	328867.31	/	/	/
	#6	39420.21	/	/	/	/	/

The serum data of #4 recipient is provided in the Figure 3B of the manuscript.
/ There were no data collected.
N The serum was not enough for qPCR analysis of HBV DNA.

* The value was below the lower limit detection (0.05 IU/mL).
The cutoff of HBeAg ≥1.0 is positive.
The value of ALT > 50 IU/mL is positive.
Mouse #6 at day two, #5 at day seven, #3 at day 14 was euthanized to collect hepatic tissue sample, respectively.
